# 4-(4-Bromo­styr­yl)-1-methylpyridinium tosyl­ate

**DOI:** 10.1107/S1600536813009227

**Published:** 2013-04-10

**Authors:** M. Krishna Kumar, S. Sudhahar, B. M. Sornamurthy, G. Chakkaravarthi, R. Mohan Kumar

**Affiliations:** a Department of Physics, Presidency College, Chennai 600 005, India; b Department of Physics, CPCL Polytechnic College, Chennai 600 068, India

## Abstract

In the cation of the title compound, C_14_H_13_BrN^+^·C_7_H_7_O_3_S^−^, the dihedral angle between the benzene and pyridine rings is 8.34 (11)°. The Br atom is disordered over two positions with site occupancies of 0.74 (2) and 0.26 (2). The mol­ecular structure is stabilized by a weak intra­molecular C—H⋯O inter­actions. The crystal structure exhibits weak C—H⋯O and π–π [centroid–centroid distance = 3.7466 (17) Å] inter­actions, forming a three dimensional network.

## Related literature

For mol­ecular compounds with non-linear optical properties, see: Bosshard *et al.* (1995 ▶); Nalwa & Miyata (1997 ▶). For similar structures, see: Krishnakumar *et al.* (2012 ▶); Okada *et al.* (1990 ▶); Sivakumar *et al.* (2012 ▶).
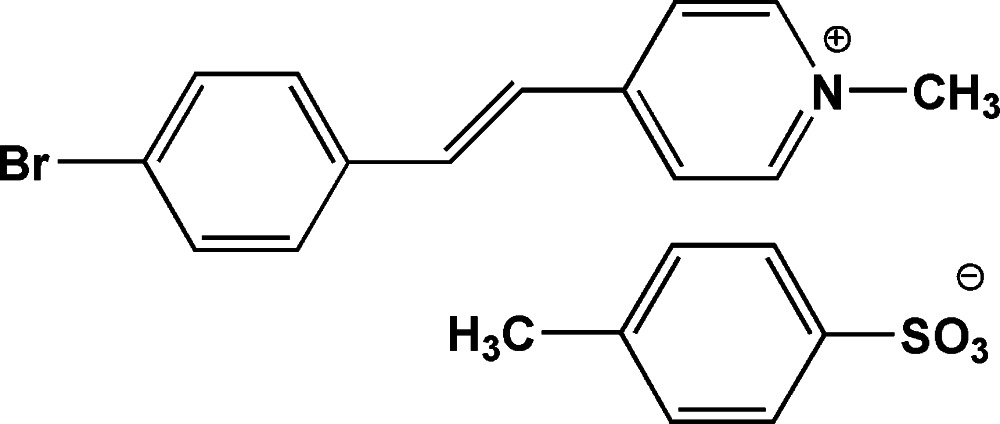


## Experimental

### 

#### Crystal data

C_14_H_13_BrN^+^·C_7_H_7_O_3_S^−^
*M* *_r_* = 446.35
Monoclinic, 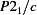

*a* = 9.0502 (2) Å
*b* = 6.4201 (1) Å
*c* = 33.9280 (7) Å
β = 94.469 (1)°
*V* = 1965.33 (7) Å^3^
*Z* = 4
Mo *K*α radiation
μ = 2.22 mm^−1^
*T* = 295 K
0.28 × 0.22 × 0.20 mm

#### Data collection

Bruker APEXII CCD diffractometer
Absorption correction: multi-scan (*SADABS*; Sheldrick, 1996 ▶) *T*_min_ = 0.575, *T*_max_ = 0.665
22764 measured reflections
5596 independent reflections
3012 reflections with *I* > 2σ(*I*)
*R*_int_ = 0.044

#### Refinement

*R*[*F* ^2^ > 2σ(*F* ^2^)] = 0.049
*wR*(*F* ^2^) = 0.130
*S* = 1.01
5596 reflections
256 parameters
1 restraint
H-atom parameters constrained
Δρ_max_ = 0.34 e Å^−3^
Δρ_min_ = −0.30 e Å^−3^

### 

Data collection: *APEX2* (Bruker, 2004 ▶); cell refinement: *SAINT* (Bruker, 2004 ▶); data reduction: *SAINT*; program(s) used to solve structure: *SHELXS97* (Sheldrick, 2008 ▶); program(s) used to refine structure: *SHELXL97* (Sheldrick, 2008 ▶); molecular graphics: *PLATON* (Spek, 2009 ▶); software used to prepare material for publication: *SHELXL97*.

## Supplementary Material

Crystal structure: contains datablock(s) global, I. DOI: 10.1107/S1600536813009227/bt6900sup1.cif

Structure factors: contains datablock(s) I. DOI: 10.1107/S1600536813009227/bt6900Isup2.hkl

Supplementary material file. DOI: 10.1107/S1600536813009227/bt6900Isup3.cml

Additional supplementary materials:  crystallographic information; 3D view; checkCIF report

## Figures and Tables

**Table 1:** Hydrogen-bond geometry (Å, °)

*D*—H⋯*A*	*D*—H	H⋯*A*	*D*⋯*A*	*D*—H⋯*A*
C10—H10⋯O1	0.93	2.57	3.415 (3)	151
C12—H12⋯O2^i^	0.93	2.39	3.247 (3)	153
C14—H14*B*⋯O1^ii^	0.96	2.53	3.438 (4)	157

Symmetry codes: (i) 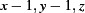; (ii) 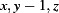.

**Fig. 1.**
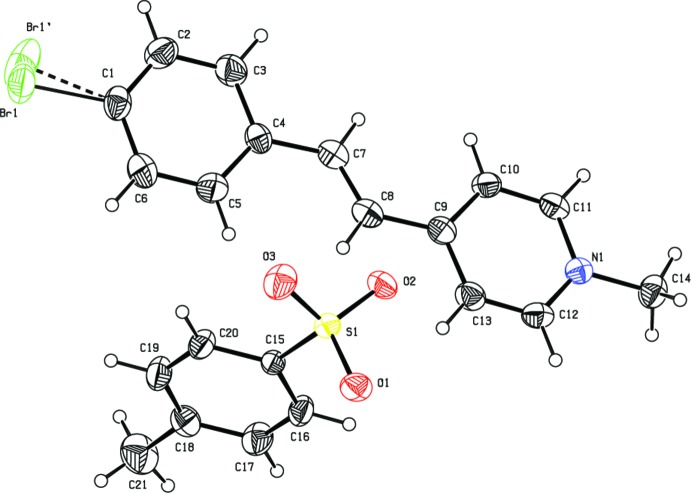
The molecular structure of (I), with atom labels and 30% probability displacement ellipsoids for non-H atoms.

**Fig. 2.**
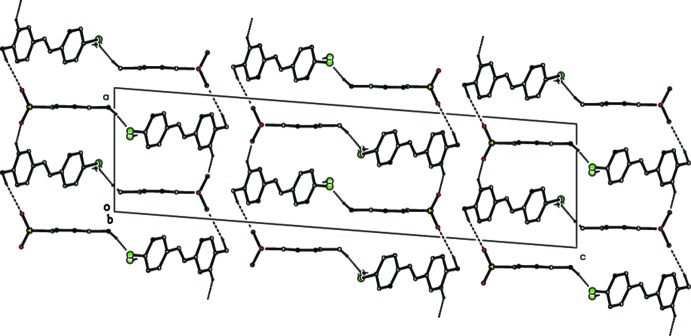
The packing of (I), viewed down b axis. Intermolecular Hydrogen bonds are shown as dashed lines. H atoms not involved in hydrogen bonding have been omitted.

## supplementary crystallographic information

### Comment

In continuation of our studies of molecular compounds with non linear optical properties which are used in optoelectronic and photonic devices (Bosshard *et al.*, 1995; Nalwa & Miyata, 1997), we herewith report the crystal structure of the title compound (I) (Fig. 1).

The asymmetric unit of the title compound consists of C_14_H_13_BrN^+^ cations and C_7_H_7_O_3_S^-^ anios. The geometric parameters of the title compound are agree well with those of reported structures (Krishnakumar *et al.*, 2012; Sivakumar *et al.*, 2012; Okada *et al.*, 1990). In the cation, the bromine atom is disordered over two positions, with the site occupancies of 0.74 (2) and 0.26 (2). The cation is planar [torision angle C4-C7=C8-C9 = 178.1 (3)°] about the double bond between the two rings in the cation. The dihedral angle between the benzene ring and pyridinium ring in the cation is 8.34 (11)°.

The molecular structure is stabilized by weak intramolecular C-H···O interactions. In the crystal structure, adjacent anions and cations are linked by weak C—H···O (Table 1 & Fig.2) and π···π [Cg1···Cg2 (x,-1/2-y,1/2+z) = 3.7466 (17)Å and Cg2···Cg1 (x,1+y,z) distance = 3.7468 (17)Å; Cg1 and Cg2 are the centroids of the rings (C9/C10/C11/N1/C12/C13) and (C1-C6), respectively] interactions.

### Experimental

The title compound was synthesized by the condensation of 4-methyl-N-methyl pyridinium tosylate, which was prepared from 4-picoline (4.65g, 5 mmol) and methyl p-toluenesulfonate (9.31g, 5 mmol), and 4-bromobenzaldehyde (9.24 g, 5 mmol) in the presence of piperidine. The single crystals suitable for X-ray diffraction were grown by slow evaporation method in room temperature.

### Refinement

H atoms were positioned geometrically and refined using a riding model with C-H = 0.93 Å and U_iso_(H) = 1.2U_eq_(C) for aromatic C-H, C-H = 0.96 Å and U_iso_(H) = 1.5U_eq_(C) for CH_3_. The components of the anisotropic displacement parameters in the direction of C4 and C7 were restrained to be equal within an effective deviation of 0.001 using DELU command in SHELXL (Sheldrick, 2008). The disorder of the bromine ligand suggests also disorder of the aromatic ring to which it is attached, but no split model for this ring could be found.

### Crystal data

**Table:**

C_14_H_13_BrN^+^·C_7_H_7_O_3_S^−^	*F*(000) = 912
*M**_r_* = 446.35	*D*_x_ = 1.509 Mg m^−^^3^
Monoclinic, *P*2_1_/*c*	Mo *K*α radiation, λ = 0.71073 Å
Hall symbol: -P 2ybc	Cell parameters from 4367 reflections
*a* = 9.0502 (2) Å	θ = 2.4–24.1°
*b* = 6.4201 (1) Å	µ = 2.22 mm^−^^1^
*c* = 33.9280 (7) Å	*T* = 295 K
β = 94.469 (1)°	Block, orange
*V* = 1965.33 (7) Å^3^	0.28 × 0.22 × 0.20 mm
*Z* = 4	

### Data collection

**Table:**

Bruker APEXII CCD diffractometer	5596 independent reflections
Radiation source: fine-focus sealed tube	3012 reflections with *I* > 2σ(*I*)
Graphite monochromator	*R*_int_ = 0.044
ω and φ scan	θ_max_ = 29.8°, θ_min_ = 2.4°
Absorption correction: multi-scan (*SADABS*; Sheldrick, 1996)	*h* = −12→12
*T*_min_ = 0.575, *T*_max_ = 0.665	*k* = −8→8
22764 measured reflections	*l* = −47→47

### Refinement

**Table:**

Refinement on *F*^2^	Primary atom site location: structure-invariant direct methods
Least-squares matrix: full	Secondary atom site location: difference Fourier map
*R*[*F*^2^ > 2σ(*F*^2^)] = 0.049	Hydrogen site location: inferred from neighbouring sites
*wR*(*F*^2^) = 0.130	H-atom parameters constrained
*S* = 1.01	*w* = 1/[σ^2^(*F*_o_^2^) + (0.0525*P*)^2^ + 0.7449*P*] where *P* = (*F*_o_^2^ + 2*F*_c_^2^)/3
5596 reflections	(Δ/σ)_max_ < 0.001
256 parameters	Δρ_max_ = 0.34 e Å^−^^3^
1 restraint	Δρ_min_ = −0.30 e Å^−^^3^

### Special details

**Table:**

Geometry. All esds (except the esd in the dihedral angle between two l.s. planes) are estimated using the full covariance matrix. The cell esds are taken into account individually in the estimation of esds in distances, angles and torsion angles; correlations between esds in cell parameters are only used when they are defined by crystal symmetry. An approximate (isotropic) treatment of cell esds is used for estimating esds involving l.s. planes.
Refinement. Refinement of F^2^ against ALL reflections. The weighted R-factor wR and goodness of fit S are based on F^2^, conventional R-factors R are based on F, with F set to zero for negative F^2^. The threshold expression of F^2^ > 2sigma(F^2^) is used only for calculating R-factors(gt) etc. and is not relevant to the choice of reflections for refinement. R-factors based on F^2^ are statistically about twice as large as those based on F, and R- factors based on ALL data will be even larger.

### Fractional atomic coordinates and isotropic or equivalent isotropic displacement parameters (Å^2^)

**Table:**

	*x*	*y*	*z*	*U*_iso_*/*U*_eq_	Occ. (<1)
Br1	0.6169 (3)	0.7372 (4)	0.03376 (7)	0.0820 (5)	0.74 (2)
Br1A	0.661 (3)	0.7520 (14)	0.0342 (2)	0.114 (3)	0.26 (2)
S1	1.20686 (7)	0.07504 (10)	0.18347 (2)	0.04224 (18)	
O1	1.07764 (19)	−0.0120 (3)	0.19985 (6)	0.0565 (5)	
O2	1.34369 (19)	−0.0096 (3)	0.20147 (6)	0.0577 (5)	
O3	1.2060 (3)	0.3000 (3)	0.18168 (7)	0.0682 (6)	
N1	0.7301 (2)	−0.7025 (3)	0.21855 (6)	0.0415 (5)	
C1	0.6600 (4)	0.5062 (5)	0.06660 (8)	0.0565 (8)	
C2	0.7976 (4)	0.4692 (5)	0.08326 (10)	0.0673 (9)	
H2	0.8749	0.5597	0.0788	0.081*	
C3	0.8230 (3)	0.2970 (5)	0.10686 (10)	0.0627 (8)	
H3	0.9181	0.2728	0.1183	0.075*	
C4	0.7112 (3)	0.1586 (4)	0.11408 (8)	0.0473 (6)	
C5	0.5724 (3)	0.2032 (5)	0.09664 (11)	0.0670 (9)	
H5	0.4941	0.1143	0.1009	0.080*	
C6	0.5463 (4)	0.3750 (5)	0.07309 (11)	0.0714 (9)	
H6	0.4514	0.4017	0.0616	0.086*	
C7	0.7449 (3)	−0.0240 (4)	0.13914 (8)	0.0504 (7)	
H7	0.8441	−0.0491	0.1469	0.061*	
C8	0.6481 (3)	−0.1545 (5)	0.15144 (9)	0.0521 (7)	
H8	0.5487	−0.1269	0.1443	0.063*	
C9	0.6818 (3)	−0.3409 (4)	0.17554 (8)	0.0455 (6)	
C10	0.8234 (3)	−0.4050 (4)	0.18908 (8)	0.0493 (7)	
H10	0.9046	−0.3240	0.1837	0.059*	
C11	0.8453 (3)	−0.5847 (4)	0.21009 (8)	0.0470 (6)	
H11	0.9411	−0.6258	0.2186	0.056*	
C12	0.5923 (3)	−0.6440 (5)	0.20655 (9)	0.0512 (7)	
H12	0.5127	−0.7259	0.2128	0.061*	
C13	0.5670 (3)	−0.4672 (4)	0.18542 (9)	0.0530 (7)	
H13	0.4700	−0.4300	0.1774	0.064*	
C14	0.7558 (3)	−0.8921 (4)	0.24243 (9)	0.0569 (7)	
H14A	0.7714	−0.8550	0.2698	0.085*	
H14B	0.8416	−0.9633	0.2343	0.085*	
H14C	0.6709	−0.9818	0.2387	0.085*	
C15	1.1957 (2)	−0.0120 (4)	0.13392 (7)	0.0364 (5)	
C16	1.1865 (3)	−0.2225 (4)	0.12499 (9)	0.0510 (7)	
H16	1.1866	−0.3207	0.1451	0.061*	
C17	1.1773 (4)	−0.2852 (4)	0.08614 (10)	0.0608 (8)	
H17	1.1720	−0.4269	0.0805	0.073*	
C18	1.1756 (3)	−0.1453 (5)	0.05514 (9)	0.0552 (7)	
C19	1.1841 (3)	0.0620 (4)	0.06475 (9)	0.0581 (8)	
H19	1.1825	0.1602	0.0446	0.070*	
C20	1.1949 (3)	0.1286 (4)	0.10329 (9)	0.0506 (7)	
H20	1.2017	0.2703	0.1088	0.061*	
C21	1.1620 (5)	−0.2217 (6)	0.01292 (11)	0.0876 (12)	
H21A	1.2117	−0.1265	−0.0034	0.131*	
H21B	1.0592	−0.2301	0.0037	0.131*	
H21C	1.2065	−0.3571	0.0117	0.131*	

### Atomic displacement parameters (Å^2^)

**Table:**

	*U*^11^	*U*^22^	*U*^33^	*U*^12^	*U*^13^	*U*^23^
Br1	0.1112 (16)	0.0651 (6)	0.0679 (8)	−0.0001 (6)	−0.0054 (9)	0.0221 (5)
Br1A	0.221 (9)	0.0675 (15)	0.056 (2)	−0.014 (4)	0.035 (3)	0.0058 (14)
S1	0.0401 (3)	0.0448 (3)	0.0418 (4)	−0.0058 (3)	0.0029 (3)	0.0025 (3)
O1	0.0419 (10)	0.0793 (14)	0.0494 (12)	−0.0104 (9)	0.0113 (9)	0.0024 (10)
O2	0.0403 (10)	0.0760 (13)	0.0552 (13)	−0.0059 (9)	−0.0076 (9)	0.0081 (11)
O3	0.0979 (16)	0.0441 (11)	0.0628 (15)	−0.0029 (11)	0.0076 (12)	−0.0069 (10)
N1	0.0467 (13)	0.0424 (11)	0.0358 (12)	−0.0019 (9)	0.0053 (10)	−0.0073 (9)
C1	0.081 (2)	0.0525 (16)	0.0361 (16)	0.0023 (15)	0.0046 (15)	−0.0002 (13)
C2	0.064 (2)	0.070 (2)	0.069 (2)	−0.0132 (16)	0.0120 (17)	0.0067 (17)
C3	0.0457 (17)	0.076 (2)	0.066 (2)	0.0034 (15)	0.0024 (15)	0.0074 (17)
C4	0.0510 (16)	0.0518 (14)	0.0388 (15)	0.0038 (12)	0.0018 (12)	−0.0070 (11)
C5	0.0480 (17)	0.073 (2)	0.079 (2)	−0.0073 (15)	−0.0014 (16)	0.0167 (18)
C6	0.061 (2)	0.076 (2)	0.075 (2)	0.0052 (17)	−0.0104 (17)	0.0167 (19)
C7	0.0467 (15)	0.0560 (15)	0.0480 (17)	0.0027 (13)	−0.0005 (13)	−0.0029 (12)
C8	0.0437 (15)	0.0581 (16)	0.0536 (18)	0.0031 (13)	−0.0022 (13)	−0.0059 (14)
C9	0.0499 (16)	0.0482 (14)	0.0382 (15)	−0.0011 (12)	0.0026 (12)	−0.0095 (12)
C10	0.0436 (15)	0.0543 (15)	0.0497 (17)	−0.0118 (12)	0.0026 (12)	0.0000 (13)
C11	0.0361 (13)	0.0595 (16)	0.0449 (16)	−0.0010 (12)	−0.0010 (11)	−0.0060 (13)
C12	0.0409 (15)	0.0594 (17)	0.0534 (18)	−0.0091 (12)	0.0052 (13)	−0.0055 (14)
C13	0.0393 (15)	0.0634 (18)	0.0561 (19)	0.0012 (13)	0.0025 (13)	0.0013 (14)
C14	0.076 (2)	0.0468 (15)	0.0483 (18)	0.0000 (14)	0.0070 (15)	0.0009 (13)
C15	0.0309 (12)	0.0393 (12)	0.0389 (14)	0.0003 (9)	0.0024 (10)	0.0049 (10)
C16	0.0661 (18)	0.0366 (13)	0.0499 (18)	0.0002 (12)	0.0017 (14)	0.0081 (12)
C17	0.082 (2)	0.0402 (15)	0.060 (2)	−0.0009 (14)	0.0030 (17)	−0.0045 (14)
C18	0.0548 (17)	0.0639 (18)	0.0465 (18)	0.0039 (14)	0.0019 (14)	−0.0039 (15)
C19	0.077 (2)	0.0534 (16)	0.0438 (18)	−0.0008 (15)	0.0046 (15)	0.0125 (14)
C20	0.0622 (17)	0.0384 (13)	0.0515 (18)	−0.0031 (12)	0.0069 (14)	0.0067 (12)
C21	0.111 (3)	0.097 (3)	0.054 (2)	0.002 (2)	0.003 (2)	−0.016 (2)

### Geometric parameters (Å, º)

**Table:**

Br1—C1	1.878 (4)	C9—C10	1.390 (4)
Br1A—C1	1.924 (9)	C10—C11	1.362 (4)
S1—O2	1.4435 (19)	C10—H10	0.9300
S1—O3	1.445 (2)	C11—H11	0.9300
S1—O1	1.4458 (19)	C12—C13	1.352 (4)
S1—C15	1.767 (3)	C12—H12	0.9300
N1—C12	1.335 (3)	C13—H13	0.9300
N1—C11	1.337 (3)	C14—H14A	0.9600
N1—C14	1.471 (3)	C14—H14B	0.9600
C1—C2	1.348 (4)	C14—H14C	0.9600
C1—C6	1.361 (4)	C15—C20	1.376 (4)
C2—C3	1.374 (4)	C15—C16	1.387 (3)
C2—H2	0.9300	C16—C17	1.375 (4)
C3—C4	1.383 (4)	C16—H16	0.9300
C3—H3	0.9300	C17—C18	1.382 (4)
C4—C5	1.376 (4)	C17—H17	0.9300
C4—C7	1.466 (4)	C18—C19	1.371 (4)
C5—C6	1.372 (4)	C18—C21	1.510 (5)
C5—H5	0.9300	C19—C20	1.372 (4)
C6—H6	0.9300	C19—H19	0.9300
C7—C8	1.304 (4)	C20—H20	0.9300
C7—H7	0.9300	C21—H21A	0.9600
C8—C9	1.468 (4)	C21—H21B	0.9600
C8—H8	0.9300	C21—H21C	0.9600
C9—C13	1.380 (4)		
			
O2—S1—O3	113.31 (13)	C9—C10—H10	119.5
O2—S1—O1	112.63 (12)	N1—C11—C10	120.5 (2)
O3—S1—O1	113.60 (13)	N1—C11—H11	119.8
O2—S1—C15	105.49 (12)	C10—C11—H11	119.8
O3—S1—C15	106.03 (12)	N1—C12—C13	120.9 (3)
O1—S1—C15	104.81 (11)	N1—C12—H12	119.6
C12—N1—C11	120.1 (2)	C13—C12—H12	119.6
C12—N1—C14	120.3 (2)	C12—C13—C9	121.5 (3)
C11—N1—C14	119.6 (2)	C12—C13—H13	119.2
C2—C1—C6	120.6 (3)	C9—C13—H13	119.2
C2—C1—Br1	122.0 (3)	N1—C14—H14A	109.5
C6—C1—Br1	117.4 (3)	N1—C14—H14B	109.5
C2—C1—Br1A	109.9 (10)	H14A—C14—H14B	109.5
C6—C1—Br1A	129.5 (10)	N1—C14—H14C	109.5
C1—C2—C3	119.5 (3)	H14A—C14—H14C	109.5
C1—C2—H2	120.2	H14B—C14—H14C	109.5
C3—C2—H2	120.2	C20—C15—C16	118.5 (3)
C2—C3—C4	122.0 (3)	C20—C15—S1	120.5 (2)
C2—C3—H3	119.0	C16—C15—S1	120.9 (2)
C4—C3—H3	119.0	C17—C16—C15	119.5 (3)
C5—C4—C3	116.5 (3)	C17—C16—H16	120.2
C5—C4—C7	123.9 (3)	C15—C16—H16	120.2
C3—C4—C7	119.6 (2)	C16—C17—C18	122.4 (3)
C6—C5—C4	121.9 (3)	C16—C17—H17	118.8
C6—C5—H5	119.1	C18—C17—H17	118.8
C4—C5—H5	119.1	C19—C18—C17	116.9 (3)
C1—C6—C5	119.6 (3)	C19—C18—C21	122.7 (3)
C1—C6—H6	120.2	C17—C18—C21	120.4 (3)
C5—C6—H6	120.2	C18—C19—C20	121.8 (3)
C8—C7—C4	125.7 (3)	C18—C19—H19	119.1
C8—C7—H7	117.1	C20—C19—H19	119.1
C4—C7—H7	117.1	C19—C20—C15	120.8 (3)
C7—C8—C9	125.9 (3)	C19—C20—H20	119.6
C7—C8—H8	117.1	C15—C20—H20	119.6
C9—C8—H8	117.1	C18—C21—H21A	109.5
C13—C9—C10	116.0 (3)	C18—C21—H21B	109.5
C13—C9—C8	119.2 (2)	H21A—C21—H21B	109.5
C10—C9—C8	124.9 (2)	C18—C21—H21C	109.5
C11—C10—C9	121.1 (2)	H21A—C21—H21C	109.5
C11—C10—H10	119.5	H21B—C21—H21C	109.5
			
C6—C1—C2—C3	0.2 (5)	C11—N1—C12—C13	0.7 (4)
Br1—C1—C2—C3	−179.6 (3)	C14—N1—C12—C13	178.3 (3)
Br1A—C1—C2—C3	−178.3 (3)	N1—C12—C13—C9	−0.1 (5)
C1—C2—C3—C4	0.2 (5)	C10—C9—C13—C12	−0.9 (4)
C2—C3—C4—C5	−0.5 (5)	C8—C9—C13—C12	178.0 (3)
C2—C3—C4—C7	179.3 (3)	O2—S1—C15—C20	−117.6 (2)
C3—C4—C5—C6	0.4 (5)	O3—S1—C15—C20	2.8 (2)
C7—C4—C5—C6	−179.4 (3)	O1—S1—C15—C20	123.3 (2)
C2—C1—C6—C5	−0.3 (5)	O2—S1—C15—C16	62.9 (2)
Br1—C1—C6—C5	179.5 (3)	O3—S1—C15—C16	−176.7 (2)
Br1A—C1—C6—C5	177.8 (4)	O1—S1—C15—C16	−56.2 (2)
C4—C5—C6—C1	0.0 (6)	C20—C15—C16—C17	0.2 (4)
C5—C4—C7—C8	−7.5 (5)	S1—C15—C16—C17	179.7 (2)
C3—C4—C7—C8	172.8 (3)	C15—C16—C17—C18	−0.5 (5)
C4—C7—C8—C9	178.1 (3)	C16—C17—C18—C19	0.2 (5)
C7—C8—C9—C13	−178.3 (3)	C16—C17—C18—C21	−178.7 (3)
C7—C8—C9—C10	0.5 (5)	C17—C18—C19—C20	0.5 (5)
C13—C9—C10—C11	1.3 (4)	C21—C18—C19—C20	179.3 (3)
C8—C9—C10—C11	−177.5 (3)	C18—C19—C20—C15	−0.8 (5)
C12—N1—C11—C10	−0.3 (4)	C16—C15—C20—C19	0.5 (4)
C14—N1—C11—C10	−177.9 (3)	S1—C15—C20—C19	−179.1 (2)
C9—C10—C11—N1	−0.8 (4)		

### Hydrogen-bond geometry (Å, º)

**Table:**

*D*—H···*A*	*D*—H	H···*A*	*D*···*A*	*D*—H···*A*
C10—H10···O1	0.93	2.57	3.415 (3)	151
C20—H20···O3	0.93	2.48	2.873 (4)	106
C12—H12···O2^i^	0.93	2.39	3.247 (3)	153
C14—H14*B*···O1^ii^	0.96	2.53	3.438 (4)	157

Symmetry codes: (i) *x*−1, *y*−1, *z*; (ii) *x*, *y*−1, *z*.
